# Does overweight affect the sagittal dimension of the posterior airway space in a non‐OSAS population? A case control study

**DOI:** 10.1002/cre2.358

**Published:** 2020-12-08

**Authors:** Federico Apolloni, Stefano Fusetti

**Affiliations:** ^1^ Maxillofacial Surgery Unit, Department of Neurosciences University of Padova Medical School Padova Italy

**Keywords:** cone beam CT, obesity, obstructive sleep apnea syndrome, overweight, posterior airway space, virtual cephalometric analysis

## Abstract

**Objective:**

The null hypothesis was that, in a non‐obstructive sleep apnea syndrome population, overweight do not reduce the antero‐posterior dimension of the posterior airway space.

**Materials and Methods:**

The author retrospectively reviewed the records of subjects evaluated at the Maxillofacial Surgery Unit, Department of Neurosciences, University of Padova Medical School, Padova, Italy, from 2016 to 2018. Only patients with complete demographic, anthropological and CBCT dataset were enrolled. OSAS patient were also ruled‐out. Enrolled patients were divided into overweight (28 cases) and non‐overweight (32 controls) groups according to the patient's Body Mass Index. Each two‐dimensional cephalometric radiography obtained from the cone‐beam computer tomography dataset was evaluated in order to measure linear and angular distances between standardized cephalometric landmarks. The two‐sample *t*‐test was the statistical test applied to compare the case and control data.

**Results:**

There were no statistical differences between the two study groups for any of the evaluated variables: the null hypothesis was accepted.

**Conclusion:**

This study showed that in a non‐obstructive sleep‐apnea population, overweight and class I obesity does not influence the airway space in the antero‐posterior dimension. Further investigation should focus on categorized overweight‐obese population. Accurate and reliable protocol for tridimensional airways assessment should be implemented.

## 1 INTRODUCTION

Different anatomical and physiological factors have been recognized as contributing factors to the narrowness of the nasopharyngeal space (Badr, 2002; Fregosi et al., 2003; Goldberg & Schwab, 1998; Hui, Xiaofeng, Jun, & Huimin, 2011; Li, Guilleminault, Riley, & Powell, 2002).

Several studies have found an increased prevalence of obesity in patients with restricted upper airways. Specifically, some authors suggested that there is a high prevalence of obesity in patients affected by obstructive sleep apnea syndrome (OSAS), especially in young and middle‐aged people; obesity is therefore considered as a risk factor for OSAS. Moreover, the contribution of obesity to the pathogenesis of OSAS is indirectly supported by the observation that either medical or surgical weight reduction produced an improvement in nocturnal sleep breathing and in daytime alertness (Arens & Marcus, 2004; Fairburn et al., 2007; Tangugsorn, Krogstad, Espeland, & Lyberg, 2000).

Several hypotheses have been formulated in an attempt to explain a possible mechanism by which obesity affects OSAS. The main theory suggests that obesity may cause a decrease in the size of the upper airway by fat deposition in surrounding tissues, which contributes to hyperplasia and soft tissue collapsibility; another hypothesis suggested that mass loading of the anterior cervical region might increase upper airway resistance with consequent airflow reduction during breathing. Obesity may, also, indirectly affect airway size by causing a decrease in lung volume (Fritscher et al., 2007; Maciel Santos, Rocha, Laureano Filho, Ferraz, & Campos, 2009).

With regard to maxillomandibular hypoplasia, a large number of cephalometric and cone beam computer tomography (CBCT) or computer tomography (CT) studies have investigated the abnormal parameters that characterized subjects affected by OSAS in relation to age, sex, race and body mass index (BMI) (Abramson et al., 2011; Maciel Santos, Laureano Filho, Campos, & Ferraz, 2011; Paoli et al., 2001; Shigeta et al., 2008).

While in OSAS population a large amount of studies correlates the decreasing patency of the posterior airway space (PAS) with obesity, in non‐OSAS population the effect of overweight‐obesity and mandibular hypoplasia on the PAS are not yet defined.

The aim of the study was to compare, in a non‐OSAS population, cephalometric airways measurements between overweight‐obese and normal weight subjects; a possible effect of obesity on the reduction of sagittal dimension of upper respiratory airways was investigated. The null hypothesis was that overweight do not reduce the sagittal dimension of the posterior airway space (PAS).

## 2 MATERIALS AND METHODS

The authors retrospectively reviewed the clinical and radiological data of all patients referred from January 1, 2016 to December 31, 2018, to the Maxillofacial Surgery Unit, Department of Neurosciences, University of Padova, for dental disease or dentofacial deformity assessments. The study was approved by the Institutional Review Board and was conducted in accordance with the principles of the Declaration of Helsinki. All the enrolled patient gave permission to process personal data for scientific purpose.

**FIGURE 1:**
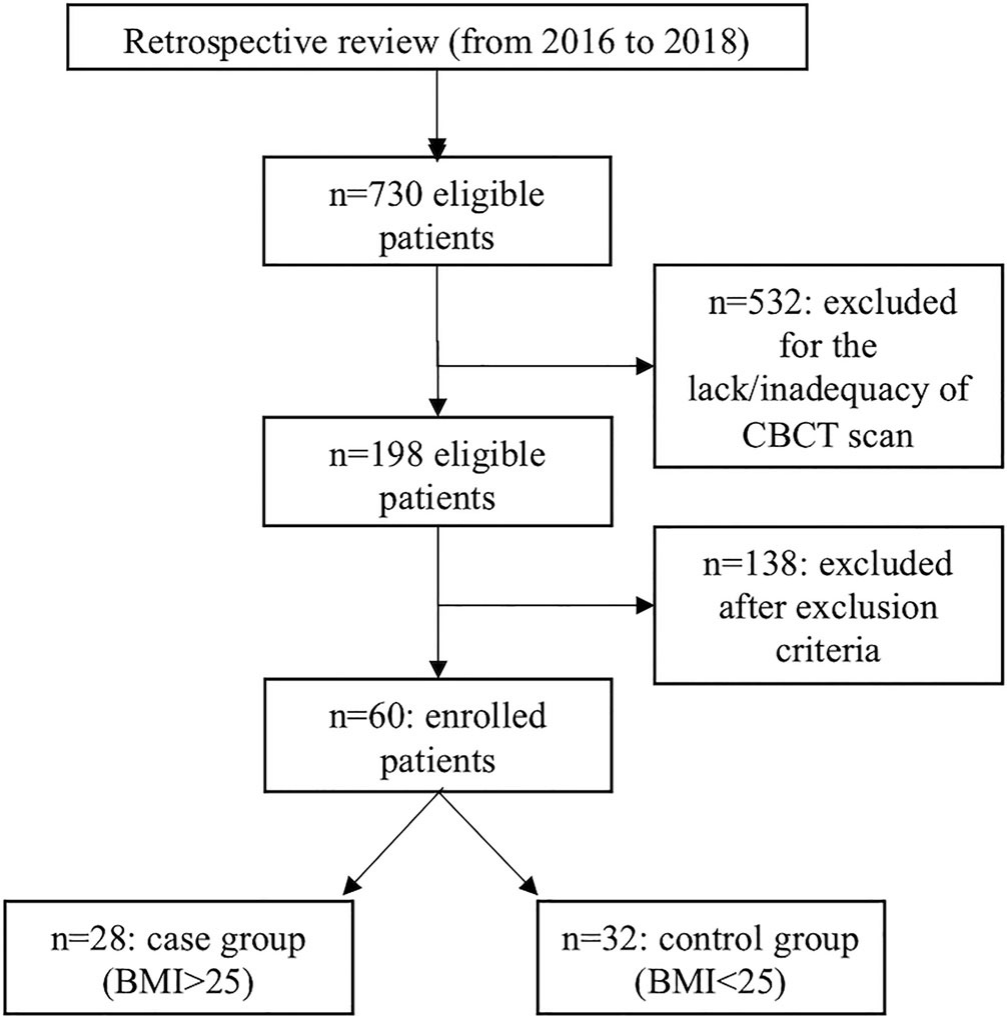
Study design

The study design is reported in Figure 1. A total of 730 medical reports were screened. One hundred ninety‐eight were found to be eligible for the study presenting adequate CT/CBCT scans and complete data regarding age, sex, height and weight.

The following exclusion criteria were applied: (a) confirmed diagnosis of OSAS; (b) subjects younger than 18 years of age, (c) craniofacial deformities, cleft palate, facial fractures, head and neck tumors, (d) previous surgical correction of dentofacial deformity. After screening with the exclusion criteria, 138 patients were excluded and 60 were included in the research.

For each enrolled patient, BMI was calculated (BMI = weight in kg/height^2^ in cm) (Kushner, 2008): patients with BMI ≥ 25 were the overweight group (cases) and those with BMI < 25 were grouped in the non‐overweight group (controls). Furthermore, patients in cases group were classified according to the accepted definition of overweight and obesity: patients with BMI between 25 and 30 kg/cm^2^ were considered overweight, with BMI between 30 and 35 kg/cm^2^ I class obesity, with BMI between 35 and 40 kg/cm^2^ II class obesity, and subjects with BMI greater than 40 kg/cm^2^ were considered III class obesity.

Each CT/CBCT dataset was imported to dedicated software with the aim of producing a lateral teleradiograph of the head (Dolphin Imaging 11.9 Premium, Chatsworth, CA). The same software was used by the same author (F.A.) to identify specific cephalometric landmarks and planes. As reported in Table 1, four cephalometric angular measurements were selected to assess maxilla‐mandibular discrepancies with reference to the cranial base; moreover, according to Riley et al., 4 linear measurements were selected based on their clinical relevance in detecting the patency of the posterior airway space (PAS) (Riley, Guilleminault, Powell, & Simmons, 1985).

**TABLE 1:** Cephalometric angular and linear measurements

Cephalometric landmarks	Planes	Angles	Linear measurements
**S (Sella)**: The midpoint of the Sella turcica	**S‐N**: a plane traced from S to N	**SNA**: Angle formed by S‐N and N‐A	**PNS‐UT**: Soft palate length
**N (nasion)**: The most posterior point of the concavity of the nasal bones	**N‐A**: a plane traced from N to point A	**SNB**: Angle formed by S‐N and N‐B	**PAS**: The distance between the base the tongue and posterior pharyngeal wall on the B‐go plane.
**Point A**: The greatest concavity of the maxilla between the maxillary dental alveolus	**N‐B**: a plane traced from N to point B	**ANB**: Angle calculated from the difference between SNA and SNB	**PAS min**: The shortest distance between the base of the tongue and the posterior pharyngeal wall
**Point B**: The deepest point in the concavity of the anterior mandible between the alveolar crest and pogonion	**Mandibular plane (MP)**: The plane from go to me	**Cervical line‐SN**: Angle formed by S‐N and cervical line. This angles changes with head flexion or extension.	**MP‐H**: The shortest distance between the hyoid bone and the mandibular plane.
**Me (Menton)**: The most inferior point of the mandibular symphysis in the midsagittal plane	**PNS to UT**: The plane from PNS to UT		
**Go (gonion)**: The point located by bisecting the angle formed by tangents to the posterior border of the ramus and the inferior border of the mandible	**B‐go**: The plane traced from B to go		
**PNS (posterior nasal spine)**: Process formed by the united projecting medial ends of the posterior borders of the palatine bones	**Cervical line**: The plane constructed parallel to the anterior surface of vertebral bodies		
**UT (uvula tip**): The most postero‐inferior point in the silhouette of the soft palate			
**H (hyoid)**: The most anterior and superior point of the silhouette of the hyoid bone			

The two‐sample *t*‐test was the statistical test applied. A *p* value <0.05 was considered significant. The STATA™ 8.1 (StataCorp LP) statistical package was used for all analyses.

## 3 RESULTS

Demographic and clinical features of the study population are reported in Table 2. Sixty patients (42 female, 18 male) aged 18 to 44 (mean 31 ± 11.3) were enrolled; the mean BMI of the population was 25.05 ± 2.5 kg/cm^2^.

**TABLE 2:** Demographic and clinical features

	Cases	Controls	*p*‐value
N. of subjects	28	32	—
Female	13	27	—
Male	15	5	—
Age (mean ± SD)	37.0 ± 13.2	25.5 ± 11.0	<0.001
BMI (kg/cm^2^)	30.8 ± 3.1	19.3 ± 1.9	<0.001

Twenty‐eight patients (13 female, 15 male) met the criteria for the case group (BMI > 25), 32 patients (27 female, 5 male) met the criteria for the control group (BMI < 25). In the case group mean age was 37.0 ± 13.2, mean BMI was 30.8 ± 3.1 kg/cm^2^; in the control group mean age was 25.5 ± 11.0, mean BMI was 19.3 ± 1.9 kg/cm^2^. Cases group had a statistically greater mean age (*p* < 0.001) and BMI index (*p* < 0.001) compared to control group; moreover, in the case group 20 patients were classified as overweight, eight patients as class I obesity, nobody as class II or III obesity.

The main cephalometric angular analysis regarding maxillo‐mandibular discrepancies is reported in Table 3: no statistical differences were found between the obese and non‐obese populations. Similarly, the cephalometric linear analysis on PAS soft tissue patency is reported in: Table 4: again, no significant p values were found between case and control populations.

**TABLE 3:** Results of angular cephalometric analysis

	Cases	Controls	*p*‐value
SNA (mean ± SD)	80.6 ± 3.1	81.9 ± 4.4	0.187
SNB (mean ± SD)	78.4 ± 5.1	78.8 ± 6.4	0.789
ANB (mean ± SD)	2.0 ± 4.1	3.1 ± 4.2	0.31
Cervical line‐SN (mean ± SD)	110.5 ± 9.2	110.3 ± 8.1	0.93

**TABLE 4:** Results of linear cephalometric analysis

	Cases	Controls	*p*‐value
PAS (mean ± SD)	10.9 ± 3.0	10.4 ± 3.4	0.547
PAS min (mean ± SD)	7.6 ± 2.8	7.8 ± 3.7	0.813
MP‐H (mean ± SD)	16.7 ± 6.3	17.4 ± 7.4	0.694
PNS‐UT (mean ± SD)	40.4 ± 5.7	39.0 ± 5.3	0.331

## 4 DISCUSSION

Many imaging techniques have been suggested for the accurate evaluation of the craniofacial skeleton and upper airway volume, such as rhinomanometry, acoustic rhinometry, magnetic resonance imaging (MRI), CT and CBCT (Arens et al., 2003; Grauer, Cevidanes, Styner, Ackerman, & Proffit, 2009; Manara et al., 2016). The authors decided to complete the planned analysis by means of a CT/CBCT dataset because of study population was chosen between healthy non‐OSAS; secondarily, the patient enrolled had been admitted to the investigating center because of the correction of dentofacial deformity and many CT/CBCTs were already performed.

In this study the airway analysis have been made by the use of 2D cephalograms because, in the authors opinion, 3D software is not a completely reliable technology so far; some authors suggest that 3D viewer softwares, despite highly reliable in their airway assessment, gave poor accuracy thus suggesting systematic errors (Hakan & Palomo, 2010); another group found a significant difference in the airway volume definition up to 42% between the semi‐automatic and the manual segmentation protocol in a widespread 3D software (De Water, Saridin, Bouw, Murawska, & Koudstaal, 2014); in another study a general underestimation of the upper airways volume have been assessed in three software packages (Chen et al., 2017). This variability seems to be correlated to the lack of a unique protocol in the dataset acquisition and processing, especially regarding threshold value selection: generally, the increase or decrease of the threshold results in a greater or smaller airway volume, respectively (Alves Jr et al., 2012); more variability is also correlated to the “partial volume effect” (Chen et al., 2017). Given the aforementioned problems in 3D PSA assessment, 2D lateral cephalogram is still considered a useful tool for the measurement of PAS size, despite the 2D limitations in 3D structures evaluation (Pirilä‐Parkkinena et al., 2011).

The airway focused cephalometric analysis showed no statistical difference in each linear measurement between case and control populations (Table 4): the original assumption that overweight would not cause reduction of PAS sagittal dimension has been accepted.

According to the accepted definition of overweight and obesity, our case group (mean BMI 30.8 ± 3.1 kg/cm^2^) considered subjects affected by just overweight and class I obesity: no class II or III patients were enrolled. This may explain why, despite the limited number of subjects enrolled, our data does not support a direct relationship between overweight‐obesity and PAS sagittal dimension: perhaps class II or III populations does have this statistical relationship; this assumption may be extended to the angular cephalometric analysis.

Some authors suggested that the oropharyngeal airway space and the hyoid position may vary according to the antero‐posterior position of the mandible (Tallgren & Solow, 1992). In order to avoid any cephalometric variability in the population, the authors assessed and compared SNA, SNB, and ANB angles between the two study groups: however, no significant differences were found between cases and control (Table 3); however, it has to be pointed out that SNA angles present a more pronounced trend toward statistical significance compare to SNB and ANB.

There is also evidence that flexion or extension of the head may influence the airway space (Hellsing, 1989). The authors assessed and compared the Cervical line‐SN angle between the two‐study group: again, there were no significant differences in the Cervical line‐SN angle between cases and controls (Table 3).

The case and the control sample in this non‐OSAS population had not significative differences in PSA and maxilla‐mandibular sagittal dimension; the only difference was the statistically greater BMI index in the case group, not enough to produce any sleep disorders. In authors opinions these findings agree with the current literature opinion: clinical and epidemiological studies show that OSAS is a multifactorial and complex disease with a strong genetic basis; on the other hand, multidisciplinary and integrated strategy is required to achieve effective and long‐lasting therapeutic success (Casale et al., 2009; Romero‐Corral, Caples, Lopez‐Jimenez, & Somers, 2010).

## 5 CONCLUSION

This investigation showed that in a non‐OSAS population, overweight and class I obesity does not influence the PAS sagittal dimension.

The study of non‐OSAS population should be encouraged to better understand the OSAS pathophysiology.

Further investigation should focus on class II‐III populations or, at least, should categorize the overweight patients according to BMI index. Moreover, future research should consider different sample age.

Novel three‐dimensional technologies should be considered in airway dimension assessment; future studies should be focused on the definition of a comprehensive and standardized 3D protocol for the evaluation of PAS. Reliability test should be performed to avoid lack of scientific significate in the results.

The main limitation of the present study is the retrospective study design: prospective multi‐institutional studies are needed to assess if any relationship does in fact exist.

## CONFLICT OF INTEREST

The authors declare no conflicts of interest.
